# Hypokalemic Periodic Paralysis as the First Manifestation of Thyrotropin-Secreting Pituitary Adenoma

**DOI:** 10.1155/2019/5913194

**Published:** 2019-10-16

**Authors:** Chatchon Kaewkrasaesin, Patinut Buranasupkajorn, Paisith Piriyawat, Sarat Sunthornyothin, Thiti Snabboon

**Affiliations:** ^1^Division of Medicine, Taksin Hospital, Medical Service Department, Bangkok Metropolitan Administration, Bangkok, Thailand; ^2^Department of Medicine, Faculty of Medicine, Chulalongkorn University, Bangkok, Thailand; ^3^Excellence Center in Diabetes, Hormone and Metabolism, King Chulalongkorn Memorial Hospital, Thai Red Cross Society, Bangkok, Thailand; ^4^Department of Neurology, Texas Tech University Health Sciences Center El Paso, El Paso, USA

## Abstract

Thyrotoxic periodic paralysis is an unusual neurological manifestation of thyrotoxicosis, and even rarer when it occurs in thyrotropin-secreting pituitary adenoma, only 6 cases having been previously reported. We describe a case of pituitary microadenoma with clinical syndromes of thyrotoxicosis complicated with hypokalemic periodic paralysis. Clinical manifestations and proposed management are discussed.

## 1. Introduction

Thyrotropin-secreting pituitary adenoma (TSHoma) is the rarest subtype among the functioning pituitary adenomas, with its prevalence less than 3% of all pituitary tumors [1]. About 80–85% of TSHomas, at diagnosis, are macroadenoma with local mass effect and mild thyrotoxicosis [2]. Herein, we report an unusual presentation of TSHoma with thyrotoxic periodic paralysis (TPP).

## 2. Case Presentation

**Figure 1:**
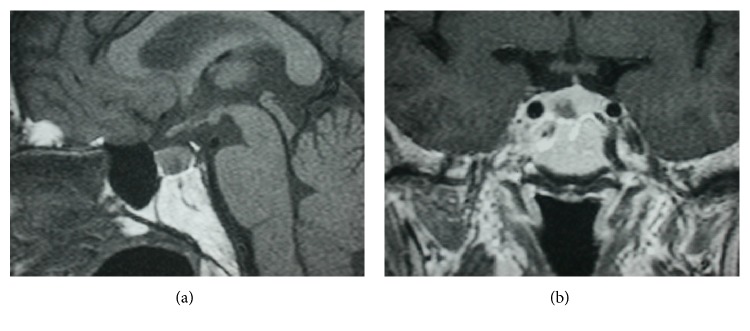
MRI pituitary gland (T1-weighted, sagittal view) showed a 6 mm hypointensity sellar lesion with irregular margin (a). On coronal postcontrast T1-weighted image, the lesion remained hypointensity while the gland showed marked homogenous enhancement (b).

A 42-year-old Thai man presented with quadriparesis upon awakening after having a high-carbohydrate dinner. His two previous episodes were mild and self-limited. He denied history of medication, alcohol use, or family history of weakness. He however noticed palpitations and 3-kg weight loss over the past 6 months. The patient was alert with tachycardia of 108/min. Neurological examination showed proximal muscle weakness grade 2/5 and the distal grade 4/5 of both extremities, while sensation and reflexes were preserved. No dysarthria, nystagmus, or cranial nerve palsy was involved. Thyroid gland was about twice the normal size without bruit or exophthalmos. Laboratory investigation revealed K^+^2.7 mEq/L and inappropriate secretion of TSH: TSH 12.96 *μ*IU/mL (0.4–4.1), FT_4_ 2.17 ng/dL (0.8–1.8), and FT_3_ 8.41 pg/dL (1.8–4.0). Other pituitary hormone levels as well as electrolytes were unremarkable (Table 1). ECG revealed sinus tachycardia with *U* wave. MRI of the pituitary gland revealed a microadenoma, 6 mm in diameter, without pressure effect on adjacent structures (Figure 1). A diagnosis of TPP from TSHoma was proposed by his dramatic recovery of muscle strength within 6 hours with rebound hyperkalemia after only 100 mEq of potassium replacement. Methimazole (15 mg/d), subcutaneous short-acting octreotide (Sandostatin 100 *µ*g three times a day) and propranolol (40 mg/d) were commenced and trans-sphenoidal surgery (TSS) was performed 4 weeks later. Pathology and immunohistochemistry confirmed the diagnosis of TSHoma, as well as a negative mutation study of the thyroid hormone receptor beta (THRB) gene. During the one-year follow-up, he was in euthyroid state and complete remission of periodic attack (Table 2).

## 3. Discussion

At diagnosis, less than 70% of TSHoma patients have thyroid manifestations, most of which are goiter or thyroid nodules, with only 20–30% of them having thyrotoxicosis symptoms [2]. We report here case of a rare neurologic-thyrotoxic concurrence, in a TSHoma patient with microadenoma.

Triad findings of TPP are transient episodes of flaccid muscle weakness, hypokalemia, and thyrotoxicosis [3]. While commonly associated with Graves' disease, it has been also described in any etiology of thyrotoxicosis. Most of TPP patients are male Asian patients despite female preponderance of Graves' disease. The characteristics of weakness are indistinguishable from other types of hypokalemic periodic paralysis which mainly involve in proximal parts of upper and lower extremities and rarely ocular, bulbar, bowel/bladder, or respiratory muscles. The frequency of attacks is variable and duration of episodes ranges from hours to several days. Severity of weakness correlates with the degree of hypokalemia. Muscle strength improves after restoration of serum potassium level and correction of thyrotoxic state. Onset is typically at night or early morning, particularly during the resting time. Myalgia has been described in some cases, while respiratory failure or fatal arrhythmia is very unusual. Predisposing factors are high-carbohydrate diet, heavy exercise, alcohol ingestion, and stress. The exact mechanism of TPP is still elusive. Intracellular shift from upregulation of Na^+^-K^+^ATPase pump is the main mechanism, whereby its activity is provoked by thyrotoxicosis state and/or various precipitating factors.

Key to prevent periodic attacks is the reestablishment of euthyroid state [3]. In case of TSHoma, administration of medications including somatostatin analog, antithyroid drug and nonselective beta-blocker prior to transsphenoidal surgery (TSS) is recommended [2, 4]. Prompt correction of serum potassium reverses muscle weakness and prevents cardiopulmonary complications such as cardiac arrhythmias. However, careful dosing of potassium replacement must be exercised to prevent rebound hyperkalemia from extracellular shift of potassium. In addition, precipitating factors should be avoided and nonselective beta blockers should be administered until a euthyroid state is achieved.

Our literature search returned only 6 case reports of TPP from TSHoma [4–9] (Table 3). Concordantly, all patients were Asian men [Japanese (2 cases), Chinese (1 case), Indonesian (1 case), Indian (1 case), and Syrian (1 case)] and most of their tumors (5 from 6 cases) were macroadenoma. Interestingly, all of them had TPP as the presenting symptom; however, their manifestations and responses to treatments, TSS with/without preoperative medications—octreotide and thionamides, did not differ from those of TSHoma without TPP.

## 4. Conclusion

We are presenting a rare case of TSHoma which manifests initially with TPP. Physician should be cognizant about the concomitant thyrotoxicosis, particularly among Asian male patients.

## Figures and Tables

**Table 1:** Laboratory data on admission.

*Hematology*	
White blood cells (cell/mm^3^)	7500
Hemoglobin	13.5
Platelets (×10^3^/mm^3^)	210

*Blood chemistry*	
Glucose (mg/dL)	95
Creatinine (0.5–1.0 mg/dL)	0.9
Sodium (135–145 mEq/L)	140
Potassium (3.4–4.5 mEq/L)	2.7
Carbon dioxide (22–29 mEq/L)	24
Calcium (8.5–10.5 mg/dL)	9.0
Phosphate (2.5–4.5 mg/dL)	2.5
Albumin (3.5–4.5 g/dL)	3.9
Magnesium (1.7–2.4 mg/dL)	1.7
CPK (0–190 U/L)	110

*Hormonal analysis*	
FT_3_ (1.6–4.0 pg/mL)	8.41
FT_4_ (0.8–1.8 ng/dL)	2.17
TSH (0.3–4.1 *µ*U/mL)	12.96
IGF-I (101–267 ng/mL)	201
Prolactin (3–25 ng/mL)	15
FSH (1.0–8.4 IU/L)	8.8
LH (1.0–10.5 IU/L)	5.6
Testosterone (290–1,300 ng/dL)	452
8 AM. Cortisol (*µ*g/dL)	10.9

*Thyroid autoantibody*	
Anti-TPO (<50 IU/mL)	34
Anti-Tg (<100 IU/mL)	56

**Table 2:** Serum potassium levels and thyroid function tests at admission, during treatment, and follow-up period.

	Admission	6 hour later^∗^	Before operation^∗∗^	12 month follow up
Serum potassium (3.5–4.5 mEq/L)	2.7	5.9	3.9	4.2
FT_3_ (1.6–4.0 pg/mL)	8.41	NA	4.6	2.3
FT_4_ (0.8–1.8 ng/dL)	2.17	NA	1.9	1.1
TSH (0.3–4.1 *µ*U/mL)	12.96	NA	8.45	3.77

^∗^After potassium chloride elixir 100 mEq.^∗∗^4 weeks later with sandostatin, methimazole, and propranolol. NA: not available.

**Table 3:** Reported cases of TSH-secreting pituitary tumor with TPP, including our case.

Age (y)/Ethnicity/Sex	Time to diagnosis (y)	Pituitary tumor (cm)	Symptoms		Serum potassium (mEq/L)	Thyroid function test					Treatment and outcome		
			Thyro-toxicosis	Goiter		FT_3_ (pg/dL)	T3 (ng/dL)	FT_4_ (ng/dL)	T4 (*µ*g/dL)	TSH (*µ*IU/mL)	TSS	Medication	Remission
27/Asian/M [4]	6	2	+	+	1.3	NA	256 (70–170)	4.1 (0.6–1.8)	23.8 (4.5–12.5)	6.4 (0.2–3.4)	+	+	+
43/Asian/M [5]	1	2	−	+	1.2	NA	430 (84–176)	3.0 (0.7–1.6)	23.3 (5.4–11.6)	4.3 (0.4–4.0)	+	+	+
44/Asian/M [6]	^∗∗^	1.5	−	−	2.0	NA	227 (100–190)	NA	14.4 (4.4–12.5)	2.10 (0.50–5.15)	+	+	+
31/Asian/M [7]	2	0.3	+	+	2.5–2.8	NA	190 (57.9–158.8)	2.1 (0.6–1.6)	NA	5.55 (0.4–4.0)	+	+	+
40/Asian/M [8]	3 month	macro#	+	−	1.2	19.9 (2.0–4.4)	NA	4.87 (0.8–1.8)	NA	56.8 (0.4–5.5)	−	+	+
28/Asian/M [9]	^∗∗^	3.1	−	+	2.5	8.0 (2.3–4.0)	NA	2.6 (0.9–1.7)	NA	7.0 (0.5–5.0)	+	+	+
42/Asian/M^∗^	^∗∗^	0.6	+	+	2.7	8.4 (1.8–4.0)	NA	2.2 (0.8–1.8)	NA	13.0 (0.4–4.1)	+	+	+

^∗^: Our case. ^∗∗^: Presenting symptom. #: Macroadenoma. +: Positive, −: Negative. NA: not available.
